# Donor-derived cell-free DNA and miRNA monitoring for the early prediction and diagnosis of liver allograft rejection and patient outcomes

**DOI:** 10.3389/fimmu.2025.1604200

**Published:** 2025-06-24

**Authors:** Judit Julian, Olga Millán, Esther Titos, Pablo Ruiz, Yiliam Fundora, Alba Díaz, Jordi Colmenero, Constantino Fondevila, Joan Anton Puig-Butillé, Mercè Brunet

**Affiliations:** ^1^ Pharmacology and Toxicology, Biochemistry and Molecular Genetics, Biomedical Diagnostic Center (CDB), University of Barcelona, Hospital Clinic of Barcelona, Barcelona, Spain; ^2^ Biomedical Research Center in Hepatic and Digestive Diseases (CIBEREHD), Instituto de Salud Carlos III (ISCII), Madrid, Spain; ^3^ Molecular Biology CORE Laboratory, Biomedical Diagnostic Centre (CDB), Hospital Clínic, Instituto de Investigaciones Biomédicas August Pi i Sunyer (IDIBAPS), University of Barcelona, Barcelona, Spain; ^4^ Liver Unit, Hospital Clinic of Barcelona, Instituto de Investigaciones Biomédicas August Pi I Sunyer (IDIBAPS), University of Barcelona, Barcelona, Spain; ^5^ Department of General and Digestive Surgery, Hospital Clínic Barcelona, Instituto de Investigaciones Biomédicas August Pi i Sunyer (IDIBAPS), University of Barcelona, Barcelona, Spain; ^6^ Pathological Department, Biomedical Diagnostic Centre (CDB), Hospital Clínic, Instituto de Investigaciones Biomédicas August Pi i Sunyer (IDIBAPS), University of Barcelona, Barcelona, Spain; ^7^ General & Digestive Surgery Service, Hospital Universitario La Paz, Research Institute University Hospital La Paz (IdiPAZ), Biomedical Research Center in Hepatic and Digestive Diseases (CIBERehd), Madrid, Spain

**Keywords:** noninvasive biomarkers, donor-derived cell-free DNA, microRNA, liver transplantation, rejection, graft dysfunction

## Abstract

**Introduction:**

The identification of noninvasive biomarkers for monitoring liver transplant (LT) recipients is crucial for the early detection of graft dysfunction and rejection. Donor-derived cell-free DNA (dd-cfDNA) and microRNAs (miRNAs) have been identified as promising biomarkers for assessing graft integrity. While the levels of dd-cfDNA have been validated for this use in kidney and heart transplantation, there are limited data regarding its potential in liver graft monitoring. Similarly, the expression levels of miRNAs, key regulators of immune responses and liver injury, have potential utility in distinguishing between rejection and other causes of graft dysfunction.

**Methods:**

In this prospective, observational study, we monitored the levels of dd-cfDNA and miRNAs by analyzing 437 plasma samples from 64 LT recipients over a 12-month period, measuring the levels of dd-cfDNA and signature miRNAs at predefined time points and during episodes of graft dysfunction. The diagnostic performance of the levels of dd-cfDNA and signature miRNAs was assessed through receiver operating characteristic (ROC) curve analysis and logistic regression models.

**Results:**

dd-cfDNA levels were significantly elevated during acute rejection (AR) episodes, with a median 3.9-fold increase over those in stable patients. A diagnostic cut-off value of 9.88% yielded an area under the ROC curve (AUROC) of 0.812, a sensitivity of 100%, a specificity of 66.7%, a positive predictive value (PPV) of 17.5% and a negative predictive value (NPV) of 100%. Interestingly, patients with cholestasis also exhibited increased dd-cfDNA levels (3.0-fold vs. stable patients), suggesting that it could serve as a potential confounder in the diagnosis of transplant rejection. Plasma miRNA analysis demonstrated significant upregulation of the expression levels of miR-155-5p, miR-122-5p, and miR-181a-5p during rejection episodes, and the incorporation of these factors improved diagnostic accuracy when combined with the level of dd-cfDNA.

**Conclusions:**

dd-cfDNA and miRNA profiling represent promising noninvasive biomarkers for diagnosing liver graft rejection and dysfunction. The combined use of these biomarkers may result in increased diagnostic accuracy, reduce unnecessary biopsies, and allow personalized immunosuppressive management. Further studies in larger cohorts are needed to validate the clinical applicability of these compounds as diagnostic biomarkers.

## 1 Introduction

Remarkable advances in the treatment of hepatic diseases and the implementation of preventive measures, the need for liver transplantation (LT) persists, however, underscoring the irreplaceable role of this technique in treating severe chronic hepatic diseases, such as cirrhosis and certain types of liver cancer (1, 2).

Despite the considerable developments in immunosuppressive therapy, approximately 20-25% of transplant recipients still develop acute rejection (AR) following grafting (3–5).

Furthermore, studies examining overall patient survival 10 years posttransplantation have indicated survival rates ranging from 50-60% (6). This is due to the development of other clinical events that may cause graft damage, such as stenosis, ischemia–reperfusion injury (IRI), viral and bacterial infections, and comorbidities associated with immunosuppressive therapies (ISTs) (7, 8).

Liver biopsy (LB) is the current gold standard for diagnosing liver graft rejection and other clinical events associated with graft dysfunction (GD), however, it is an invasive and costly procedure that involves inherent risks such as pain, bleeding, intrahepatic or subcapsular hematomas, and, in rare cases, severe complications requiring hospitalization. Moreover, despite extensive efforts to standardize procedure variability through various guidelines (9), complications still arise (10, 11) Owing to these limitations, there is a need to identify novel noninvasive biomarkers that can be used to diagnose rejection and predict graft evolution.

Donor-derived cell-free DNA (dd-cfDNA) has recently been identified as a potential biomarker of rejection in transplant patients. Organ transplantation also involves genome transplantation, and thus the donor genome could theoretically be used as a direct control for assessing the health or proper functioning of the graft. Robust clinical evidence has demonstrated that dd-cfDNA levels are a reliable biomarker for assessing graft integrity and for detecting or excluding episodes of rejection. In fact, plasma dd-cfDNA levels have been clinically validated as a biomarker for monitoring the health of kidney, heart and lung transplants (12–18). Moreover, the determination of dd-cfDNA levels also allows adjustments to be made to immunosuppressive therapy in transplant recipients. In a review of studies on kidney transplantation, *Oellerich* et al. (19), noted the utility of dd-cfDNA as a biomarker for distinguishing between patients experiencing rejection and those with overimmunosuppression, thus allowing the implementation of a more personalized treatment approach.

Different techniques can be used to analyses dd-cfDNA, such as shotgun sequencing, droplet digital PCR (ddPCR), and targeted next-generation sequencing (NGS) (20, 21). Each of these methods has advantages and disadvantages in terms of, for example, cost or processing time; nevertheless, among these techniques, targeted NGS has gained the greatest attention owing to its use of single nucleotide polymorphisms (SNPs) for precise genotyping, enabling accurate calculation of the percentage of dd-cfDNA in plasma as well as the development of scalable and cost-efficient workflows for clinical implementation. Additionally, dd-cfDNA can be expressed in different ways, such as the percentage (graft cfDNA/total cfDNA) or as an absolute quantity in copies/milliliter (22–25). It is still unclear, however, which of the two methods of expression yields better results. In the present study, we use the percentage, as the kit from which we obtained the results only provides the results in this format.

dd-cfDNA has been widely studied in renal transplantation, where its threshold for diagnosing rejection is well established and clinically used (26–28). In a large international study involving over 2000 patients, Loupy et al. (26) confirmed that higher dd-cfDNA levels correlate with all types of renal rejection and improve predictive accuracy beyond that of standard monitoring.

However, its use in liver transplantation (LT) has been less explored, with few studies and small patient cohorts (29–31). Our research group published a preliminary study with a small sample size and based on a different methodology (short tandem repeats); the results demonstrated that dd-cfDNA could detect liver graft rejection even 1–2 weeks before clinical signs and decreased after treatment (32). The current goal is to assess the usefulness of dd-cfDNA for ongoing monitoring in LT patients, to help diagnose rejection or other complications [e.g., infections such as cytomegalovirus (CMV), biliary issues, IRI] and tailor immunosuppressive therapy accordingly.

Additionally, microRNAs (miRNAs) are being investigated as noninvasive biomarkers of transplant rejection and graft function. miRNAs regulate immune responses and have been linked to both T-cell-mediated rejection (TCMR) (33–37) and antibody-mediated rejection (ABMR) (38–40) in kidney and liver transplantations. Our group previously identified a miRNA signature (miRNA-155-5p, miRNA-122-5p, and miRNA-181a-5p) in adult liver recipients that reflect both liver damage and the immune response and have the potential to guide adjustments to IST (41–43).

The use of biomarkers that can be analyzed in minimally invasive samples, such as plasma, would enable monitoring of graft function in transplant patients and thus real-time assessment of the status of each recipient, allowing adjustments to be made to the IST dose according to the patient’s profile. ISTs are essential for preventing transplant rejection, but long-term use can lead to comorbidities such as nephrotoxicity, cancer, cardiotoxicity, and diabetes, among others. Therefore, distinguishing patients at risk of rejection from those who are not—and therefore could undergo IST dose reductions to prevent these comorbidities is crucial.

Currently, there is a lack of robust data regarding the role of dd-cfDNA and miRNAs separately as noninvasive early biomarkers for assessing the risk of rejection and patient outcomes in liver transplantation, with only a few studies available and some controversy in the findings (24). Furthermore, no published evidence that combines this biomarker with miRNAs has been reported.

The aim of this study was to assess the potential of dd-cfDNA monitoring in the detection of GD (primarily rejection) in LT recipients. Furthermore, we aimed to establish a dd-cfDNA threshold in LT patients to effectively distinguish between those who could experience rejection and those who might not. In addition, we aimed to develop a diagnostic score integrating dd-cfDNA levels and a miRNA signature to improve the identification of patients at risk of graft rejection and dysfunction.

## 2 Materials and methods

### 2.1 Patients and methods

We conducted a prospective, observational cohort study from April 2022 to May 2024. A total of 155 adult patients underwent LT at our center (Hospital Clínic Barcelona) during the study period. All patients scheduled for transplantation were informed about the study, and 70 individuals consented to participate by signing the informed consent form. However, six of these patients were excluded from the study for various reasons: three patients died prior to transplantation, one patient passed away in the operating room, and two patients experienced early postoperative mortality due to septic shock and associated multiorgan failure. All patients were followed up during the first year after LT. Prior to participation, all patients provided informed consent, and the study was approved by the Institutional Review Board (IRB) under the assigned numbers (HCB/2019/0258 and HCB/2021/0751).

### 2.2 Immunosuppression

IST regimens were defined according to the Child–Pugh classification of the pretransplant liver status. Patients with Child–Pugh class A cirrhosis or other conditions associated with a relatively low risk of renal injury received dual IS therapy (starting within 24 hours after LT) consisting of tacrolimus (TAC) with target trough levels of 8–10 ng/mL and a tapering dose of corticosteroids to be withdrawn 6 months after LT.

Patients with Child–Pugh B or C cirrhosis, those who underwent transplantation due to acute liver failure or retransplanted patients, received induction therapy with a single dose of basiliximab (20 mg) immediately after LT. Then, triple IST was started involving mycophenolate mofetil (MMF) 2000 mg daily, TAC starting on day 5 after LT with a target trough level of 5–8 ng/mL, and a tapering dose of corticosteroids to be withdrawn 6 months after LT. In all of these patients, the MMF dose was reduced to 1500 mg daily 1 month after LT. A low dose of prednisone (2.5–5 mg) was maintained long-term in patients who underwent transplantation due to autoimmune hepatitis either in the double or triple IS therapy groups.

### 2.3 Study design and sample collection

All LT recipients were managed by transplant hepatologists according to standardized protocols throughout the follow-up. Study visits for liver function testing (LFT), pharmacokinetic monitoring and plasma collection were performed at day 1 (V1), weeks 1 (V2) and 2 (V3) and months 1 (V4), 3 (V5), 9 (V6) and 12 (V7) (for a total of 7 plasma samples per patient). An additional peripheral blood sample was collected prior to LT for miRNA expression analysis and genomic DNA extraction, the latter of which was subsequently used for evaluating dd-cfDNA levels. If a biopsy was deemed necessary by the clinician to prove rejection, the visit was also used to collect a blood sample from the patient (VEC). For this reason, some patients have up to 8 or even 9 samples. Patients were not excluded if they missed one of the per-protocol visits (V1-V7). Clinical events that occurred between visits were grouped into the closest visit for the purpose of statistical analysis. All patients were anonymized via numerical identifiers assigned to each patient and sample, which were used for sample tracking throughout the study. All biomarker assessments for evaluating graft progression were conducted at the laboratories of the Hospital Clínic de Barcelona. Specifically, dd-cfDNA was analyzed at the Core Laboratory of Molecular Biology, whereas miRNA determination and IS treatment monitoring were carried out at the Pharmacology and Toxicology Laboratory (CDB).

GD was diagnosed in patients who exhibited abnormal LFT results, defined as serum levels of aspartate aminotransferase (AST), alanine aminotransferase (ALT), or bilirubin exceeding twice the upper limit of normal during routine follow-up laboratory monitoring or if these parameters failed to decrease within the first two weeks following LT. Furthermore, an abdominal ultrasound was performed to exclude vascular or biliary complications that could account for these biochemical abnormalities. Patients diagnosed with GD not caused by biliary or vascular complications, subsequently underwent LB to rule out graft rejection. Additionally, CMV infections were closely monitored. For the first two months after LT, all patients had their plasma CMV viral load measured weekly and then at least monthly for six months. CMV infection was defined by a CMV DNA level >1000 copies/mL and was recorded if it coincided with a study visit.

### 2.4 Liver biopsy

All biopsies were reviewed by a qualified pathologist according to the Banff Working Group criteria (44) to determine the diagnosis and severity of rejection. Other potential causes of GD identified in the biopsies, in addition to rejection, were also documented. Biliary stricture (BS), a significant and common occurrence in the study population that can lead to abnormal LFTs, was also noted. Patients suspected of BS underwent either magnetic resonance cholangiopancreatography (MRCP) or endoscopic retrograde cholangiopancreatography (ERCP) for diagnostic confirmation. This event was recorded during the study visit prior to any BS treatment.

### 2.5 Pharmacokinetic monitoring

The TAC trough concentrations were assessed during the first week, on day 15, and at the 1st, 2nd, 3rd, 9th, and 12th months following LT. Whole-blood TAC concentrations were quantified with a Tacrolimus-CMIA-Architect assay (Abbott, Wiesbaden, Germany) in accordance with the manufacturer’s guidelines. Fresh, nonfrozen samples were analyzed daily. The laboratory’s adherence to LGC Standard Proficiency Testing is ensured through its participation in the United Kingdom External Analytical Quality Assessment Service.

### 2.6 Genomic DNA and cell-free DNA extraction

Genomic DNA (gDNA) was extracted from 1 ml of peripheral blood, which was collected before the transplantation procedure, with a MagNa Pure 96 DNA and Viral NA Large Volume Kit in a MagNA Pure 96 Instrument (Roche Diagnostics, Basel, Switzerland). In cases where a pretransplant blood sample was unavailable (n=5), a saliva sample was collected a few days after transplantation for gDNA extraction with a Maxwell^®^ CSC nucleic acid extractor instrument (Promega Corporation, Madison, WI, USA).

Plasma cfDNA was extracted from peripheral blood samples collected directly by venipuncture into two 10 ml Cell-Free DNA BCT tubes (Streck, LaVisa, NE, USA). The plasma was isolated according to a two-step centrifugation protocol: the first centrifugation at 1.600 × g for 20 min and the second at 16.000 × g for 10 min. The isolated plasma was stored at -80°C until required for cfDNA extraction. cfDNA was then extracted from 5 ml of plasma using the QIAmp Circulating Nucleic Acid Kit (Qiagen, Düsseldorf, Germany) according to the manufacturer’s instructions. Eluted cfDNA was quantified using the Qubit™ High Sensitivity DNA Assay (Thermo Fisher Scientific, Waltham, MA, USA) and stored at -20°C for subsequent NGS analysis.

### 2.7 dd-cfDNA quantification with NGS

dd-cfDNA levels were measured in our cohort and at the established times using the AlloSeq cfDNA Kit (CareDx, San Francisco, CA, USA). Briefly, 10 nanograms of cfDNA or gDNA were extracted to quantify the dd-cfDNA levels in LT recipients. Library preparation was performed with the Alloseq cfDNA Kit according to the manufacturer’s instructions, and sequencing was carried out on a MiSeq platform (Illumina, San Francisco, CA, USA) using the MiSeq v3–150 cycle sequencing kit. Data analysis was carried out with AlloSoft v2.2.1 software (CareDx) with the recipient’s genotype from the pretransplant gDNA sample. dd-cfDNA expression levels were automatically calculated and reported by the software as a percentage of the total cfDNA present in the sample.

### 2.8 Plasmatic miRNA analysis

At the time of the clinical visits and biopsies, plasma miR-155-5p, miR-122-5p and miR-181a-5p expression was measured by quantitative real-time PCR (qPCR) using a LightCycler 480 Real-Time PCR System (Roche, Basel, Switzerland). Blood samples (3 ml) were collected in EDTA-K3 tubes at the pretransplantation visit and at each visit after LT according to the study design. Blood samples were obtained prior to immunosuppressant administration (predose); at points concurrent with rejection episodes, the samples were collected before any treatment changes were made. After centrifugation (within 2 hours) at 3,000 rpm for 10 min, the plasma was collected and stored in RNase-free tubes at -70°C for batch analysis.

Plasma miRNA expression was analyzed as previously described by our group (45). Briefly, total RNA was purified from patient plasma with miRCURY™ RNA Isolation Kits–Biofluids (Qiagen, Hilden, Germany) according to the manufacturer’s instructions and reverse transcribed into cDNA. qPCR was performed with a miRCURY LNA SYBR Green PCR Kit (Qiagen ID: 339347, Polyadenylation and cDNA Synthesis System; Qiagen, Hilden Germany). The amplification curves were analyzed using Roche LC Software for determining Cq by the second derivative method. ΔCq was calculated as the difference in Cq values between the miRNA target and the reference control (miR-103a-3p and miR-191-5p), following the manufacturer’s instructions; relative expression levels of target miRNAs within a sample were then determined according to the formula 2^(–Δ^ *^Cq^* ^)^, where high values corresponded to higher expression levels.

### 2.9 Statistical analysis

Statistical analysis was performed with SPSS software, version 27.0 (SPSS Inc., Chicago, IL, USA), and R studio (R studio Inc., Boston, USA) was used for logistic regression.

To compare the different groups, we used the Mann–Whitney U test, a nonparametric test, for data that did not follow a normal distribution. For categorical variables, we used the chi-square test for between-group comparisons. All data are presented as the median ± standard deviation (SD) or interquartile range (IQR). A p value ≤ 0.05 was considered to indicate statistical significance. As we had 14 rejection samples, it was decided to merge them into a single group regardless of the visit at which rejection occurred to facilitate comparisons with the control groups. Therefore, only the diagnostic capability and not the predictive capacity of the biomarker (dd-cfDNA) was evaluated. The diagnostic performance of the biomarkers was assessed by estimating the area under the receiver operating characteristic (ROC) curve (AUROC) and its 95% confidence interval (95% CI). The cut-off points were defined as those that maximized the Youden index. We used logistic regression to evaluate the predictive ability of the different biomarkers, using the dichotomous dependent variable of the presence or absence of rejection.

## 3 Results

### 3.1 Rejection and graft dysfunction episodes

**Figure 1:**
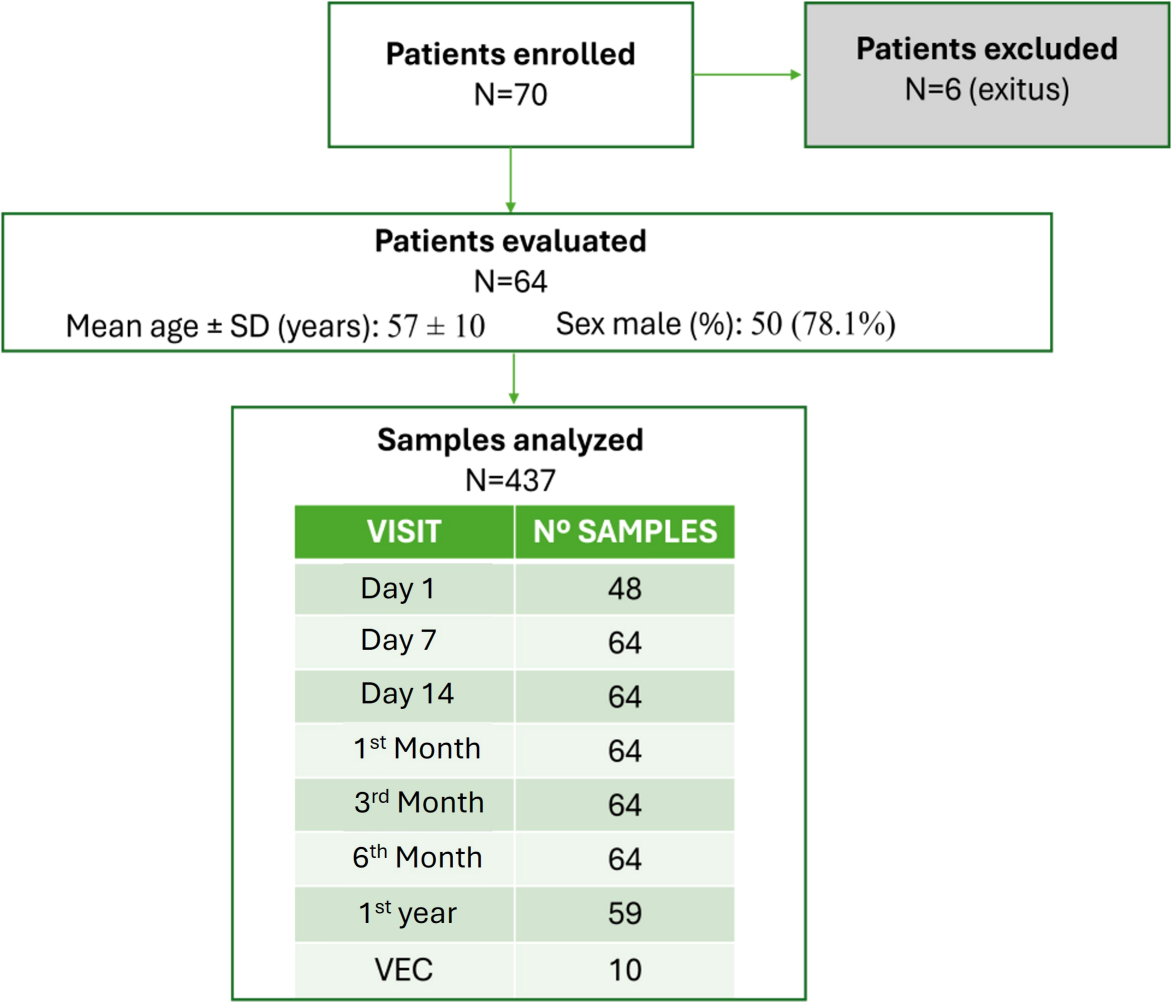
Flowchart displaying patient enrolment and sample analysis.

We evaluated the dd-cfDNA and miRNA levels in 437 samples from 64 patients over a period of 12 months. **Figure 1** shows the total number of samples available at each visit. On average, each patient made 6.8 visits. The clinical, demographic, and laboratory data collected for the study are outlined in **Table 1**. Most of the patient cohort consisted of males (78.1%), and the median age was 57 years. A total of 97.2% of the participants were Caucasian, 1.4% were of Maghrebi origin, and 1.4% were Asian. Predominant indicators for LT included alcohol-related cirrhosis (43.7%) and hepatitis C virus infection (17.2%), with hepatocellular carcinoma accounting for 15.6% of the cases. All grafts were retrieved from deceased-donor liver transplants (DDLTs); most originated from individuals who experienced brain death (48.4%), the median donor age was 52 years, and the median cold ischemia time (CIT) was 444 minutes. During the follow-up, 46 episodes of clinical events were reported.

**Table 1:** Demographic and clinical characteristics of all the patients.

	Total (64)	Rejectors (13)	Non-rejectors (51)	P-value
Recipient Sex (male)	50 (78.1%)	11 (84.6%)	34 (66.6%)	0.60
Recipient age(years)	57 ± 10	57± 10	57± 10	0.73
Donor Age (years)	52 ± 14	54,5 ± 14	53,7 ± 14	0.80
Prim. Disease				
Alcohol	28 (43.7%)	4(30.7%)	24(47%)	0.11
HCV	11 (17.2%)	4 (30.7%)	7(13.7%)	0.93
HBV	7 (10.9%)	2(15.4%)	5(9.8%)	0.80
Autoimmune	7 (10.9%)	2(15.4%)	5(9.8%)	0.80
Cholestatic	1 (10.9%)	1 (7.7%)	–	0.07
Cryptogenic	1 (1.5%)	–	1(2%)	0.56
NAFLD	3 (4.6%)	–	3 (5.8%)	0.31
Polycystosis	3 (4.6%)	–	3 (5.8%)	0.31
Others	10(15.6%)	2 (15.4%)	8 (15.7%)	0.71
HCC	10 (15.6%)	5(38.4%)	5 (5.8%)	0.05
DBD	31(48.4%)	8(61.5%)	23(45.1%)	0.54
Ischemia time (min)	444 ± 212	464 ± 208	442,45 ± 216	0.37
IS protocol				
Double	5 (7.8%)	1 (7.7%)	4 (7.8%)	
Triple	59 (92.2%)	12 (92.3%)	47 (92.2%)	

HCV, hepatitis C virus; HBV, hepatitis B virus; HCC, hepatocellular carcinoma; DBD, donor after brain death.

Biopsies confirmed that 14 of the clinical events were acute graft rejection events (8 moderate and 6 mild); of these, 13 were TCMRs, and one was plasma cell-rich rejection (PCRR). A total of 13 patients experienced rejection (one patient experienced two episodes of TCMR). Other patients experienced GD but not rejection: 15 with biliary stenosis, 9 with active CMV infection (>1000 cp), 5 with IRI, and 3 with nonspecific inflammation. The clinical characteristics of the groups were similar. Most acute rejections occurred within the first month (12/14), as did all IRIs (5/5) and most CMV (7/9) infections. However, most biliary obstructions occurred after the first month (13/15).

The concentrations of TAC (Cmin) were analyzed in both the stable patient group and the rejection group, yielding median values during the entire follow-up of 5.9 ng/mL and 7.0 ng/mL, respectively. No statistically significant differences were observed between the groups (p value = 0.22).

All acute rejections were resolved with treatment, and no grafts were lost due to rejection.

### 3.2 dd-cfDNA levels in stable functioning patients

**Figure 2:**
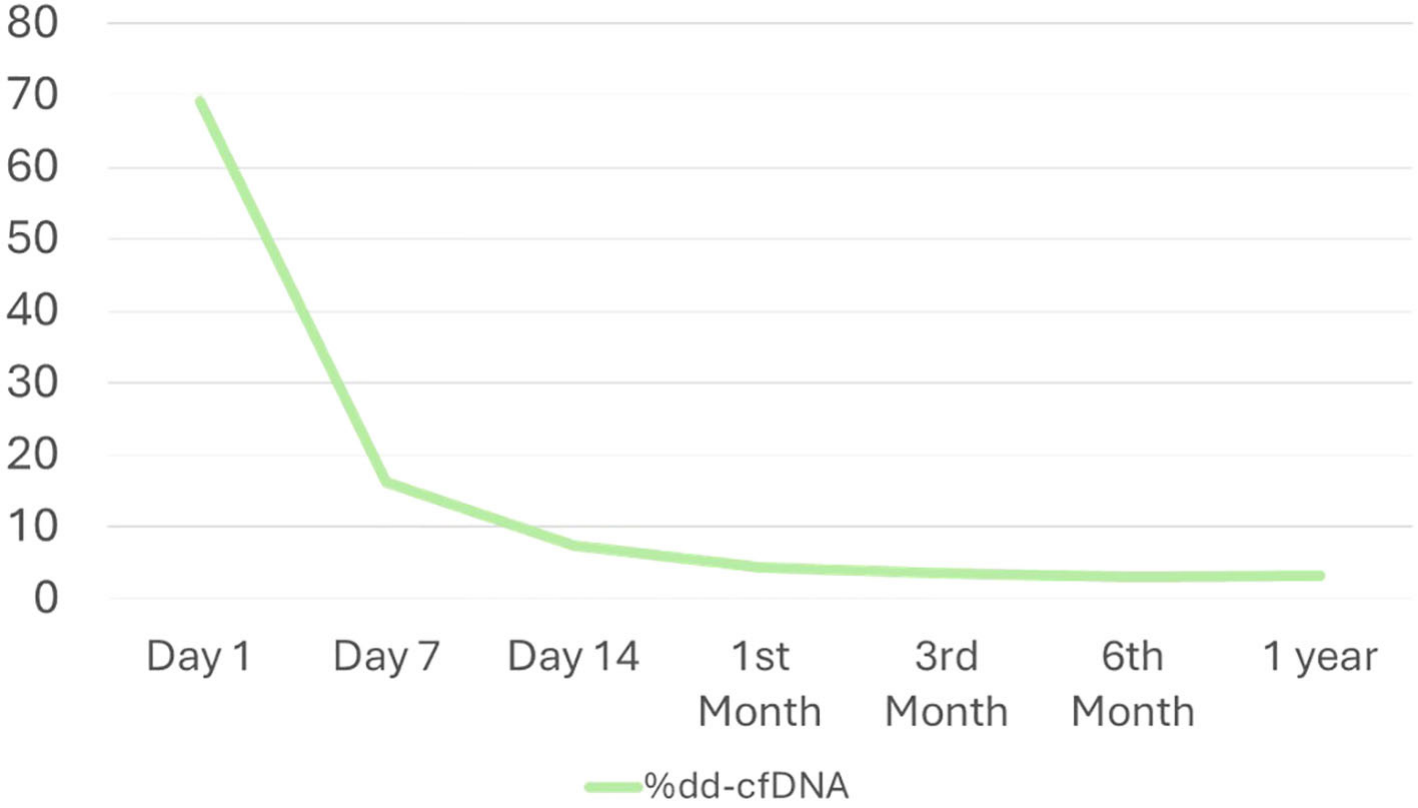
Temporal evolution of dd-cfDNA levels during the follow-up period.

The median dd-cfDNA levels of the stable patients without any clinical events at each visit are shown in **Table 2**. On posttransplant day 1 (V1), the dd-cfDNA levels were highly elevated, reflecting the damage the transplanted organ experienced during the IRI process. The values of the biomarker decreased rapidly after the first week posttransplant in these patients, reaching baseline levels (<10%) from the second week onwards, which were maintained throughout the year (**Figure 2**). During the initial visits, we observed a wide IQR; however, by the second week (V3), the IQR had significantly narrowed.

**Table 2:** Median values and interquartile ranges (IQRs) of the expression level of dd-cfDNA across different patient visits.

Visit	Median (%)	IQR
Day 1(V1)	68.4	40.1
Day 7(V2)	16.6	17.6
Day 14 (V3)	7.67	11.6
1^st^ month (V4)	4.33	6.7
3^rd^ month(V5)	3.02	4.5
6^th^ month(V6)	3.09	4.1
1^st^ year(V7)	3.35	2.83

### 3.3 dd-cfDNA levels in graft dysfunction patients

Significant differences were found in dd-cfDNA levels between samples from patients with TCMR (median value= 25.4) and nonrejection (median value= 6.5) patients (p value < 0.01) (Mann–Whitney U test = 0.937). When comparing each visit individually, except for the first visit (the day of transplantation), all subsequent visits showed significant differences in the %dd-cfDNA value between the stable patient group and the group experiencing rejection (7 days, p value = 0.018; all following visits, p value < 0.01). Patients with TCMR had a median %dd-cfDNA 3.9 times higher than the stable group. We also evaluated potential differences between mild and moderate acute rejection, but no significant differences were found (p value = 0.176).

**Figure 3:**
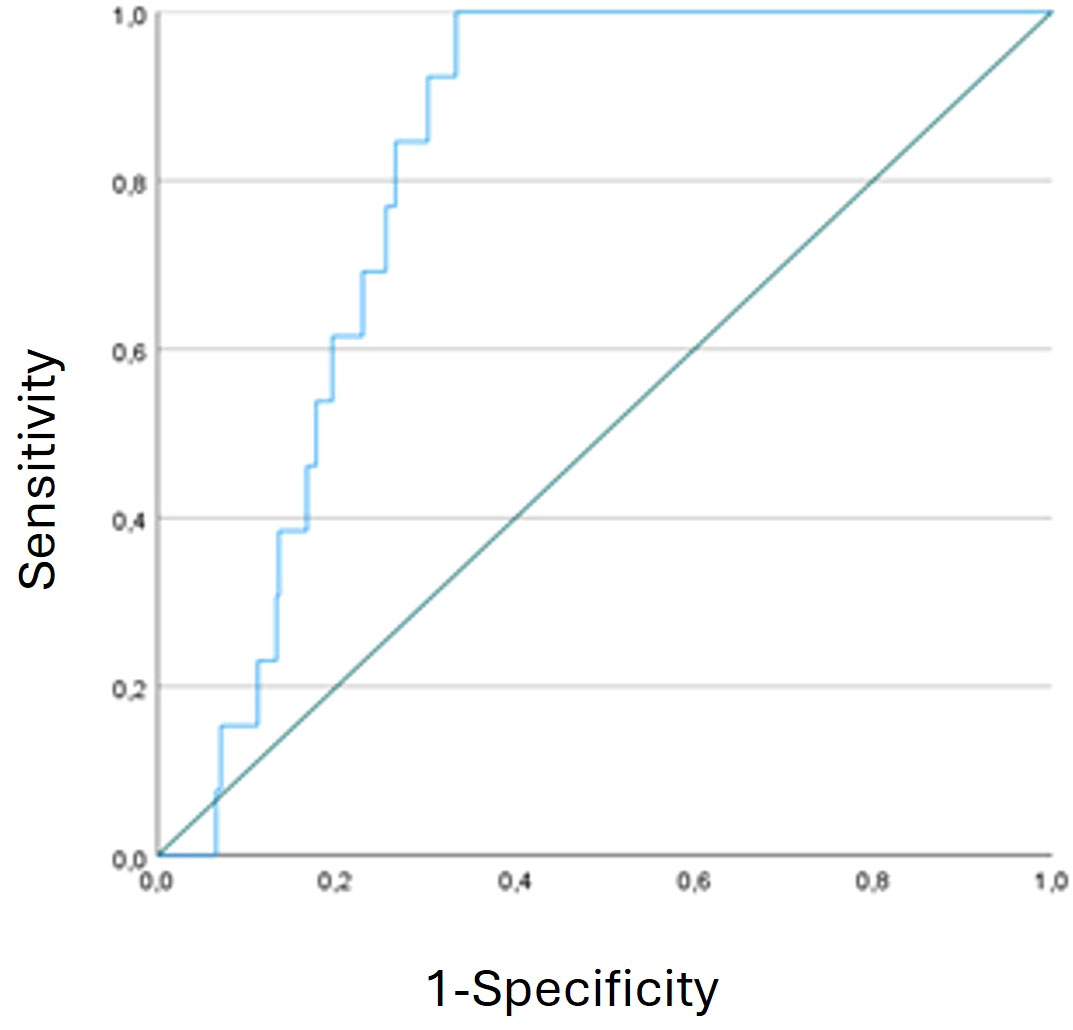
Receiver operating characteristic (ROC) curve analysis of %dd-cfDNA with diagnostic capability.

The AUROC for distinguishing between TCMR patients and stable patients at the time of diagnosis was 0.812 (95% confidence interval [95% CI], 0.757–0.868). The diagnostic sensitivity of %dd-cfDNA was 1, and the specificity was 0.667 at a threshold value of 9.88%, yielding a PPV of 17.5% and an NPV of 100%. (**Figure 3**) The %dd-cfDNA values fell to baseline levels after successful rejection treatment. (**Supplementary Figure S1**).

**Figure 4:**
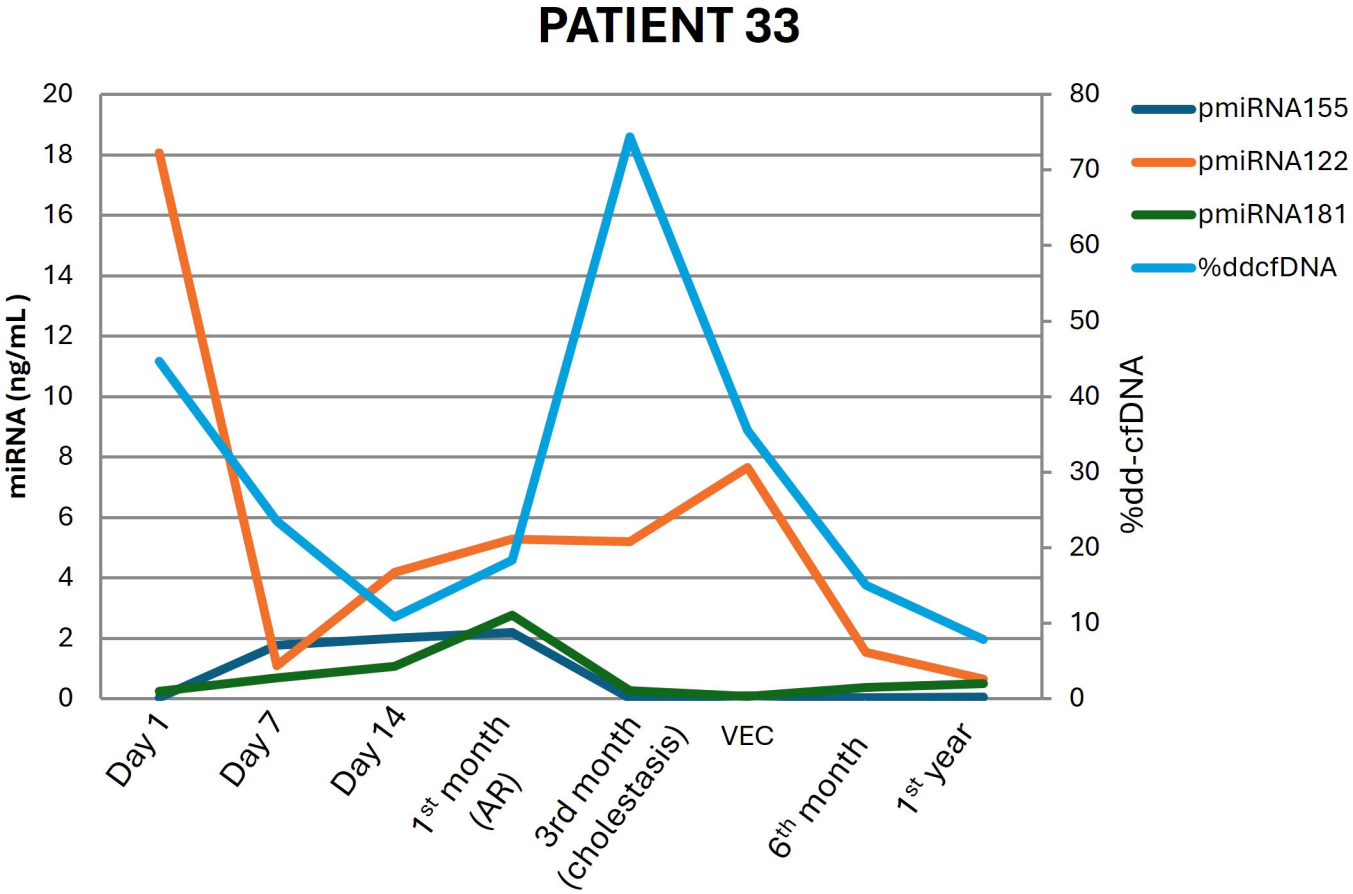
Donor-derived cell-free DNA (dd-cfDNA) and miRNA signature dynamics over time in patient 33, who underwent liver transplantation and experienced rejection at the 1^st^ month and biliary stenosis at the 3^rd^ month.

**Figure 5:**
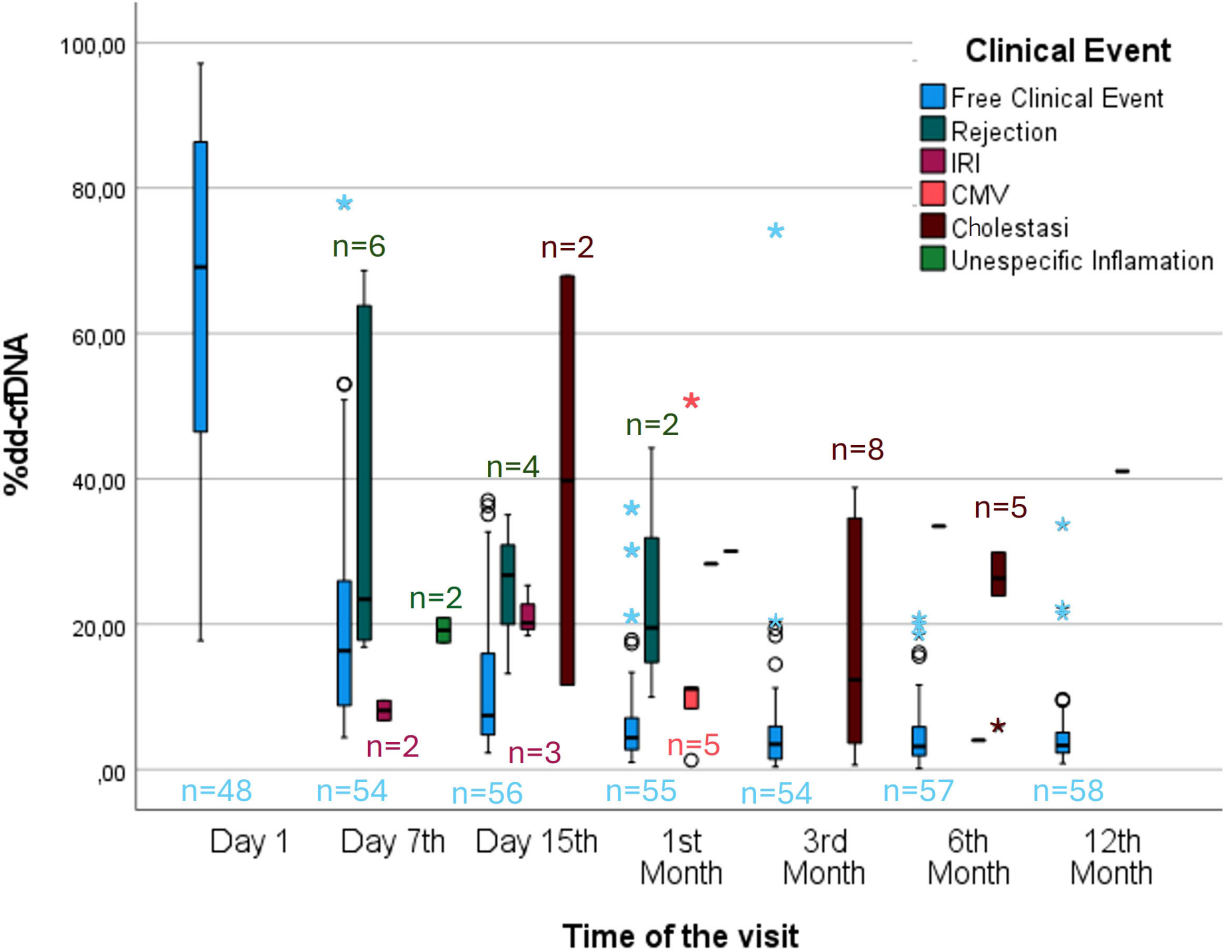
Monitoring of %dd-cfDNA after liver transplantation. Box plot showing the plasma %dd-cfDNA values between stable patients and patients who experienced a clinical event. ◯→ Represents moderate outliers, meaning values that are between 1.5 and 3 times the IQR above or below the quartiles. *→ Represents extreme outliers, meaning values that are more than 3 times the IQR above or below the quartiles.

When we compared the %dd-cfDNA values between the different groups of nonrejection patients with clinical events (CMV, IRI, biliary stenosis, nonspecific inflammation) and stable patients, we found significant differences only between the biliary stenosis group and the stable group (p value = 0.014), the former of whom demonstrated a median %dd-cfDNA value 3.0 times higher than that of the latter. This finding suggests that biliary stenosis could act as a confounding factor in cases where the established cut-off value is exceeded. (**Figure 4**) The %dd-cfDNA values grouped by visit for the different patient groups are represented in **Figure 5**. The outlier values observed within the category of patients without clinical events mostly corresponded to patients who developed a clinical event in subsequent visits. For example, some of the samples were from patients who were diagnosed with biliary stenosis in subsequent visits (starting from 7 days post-LT).

### 3.4 miRNAs in graft dysfunction patients

**Figure 6:**
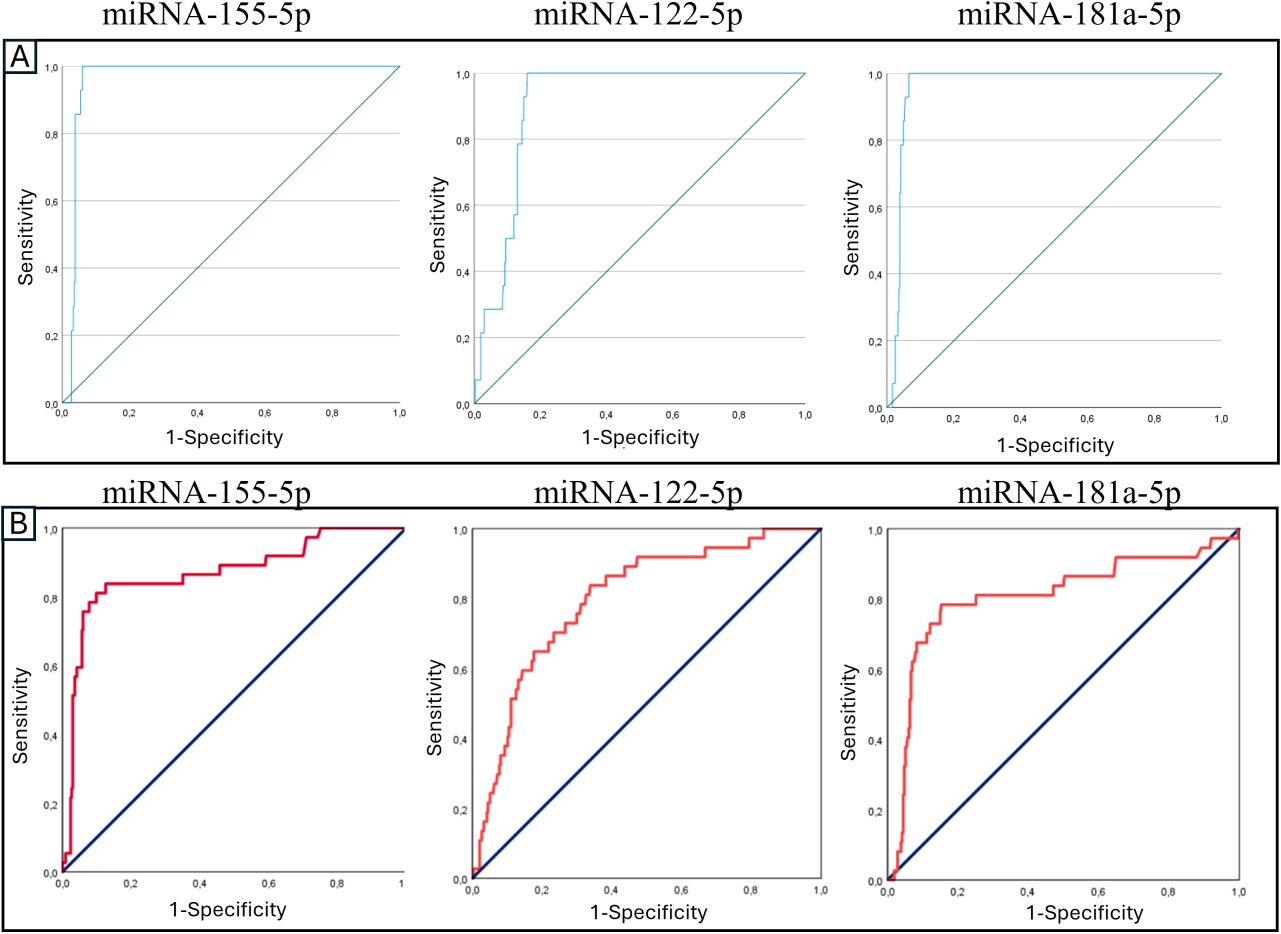
Receiver operating characteristic (ROC) curve analysis of the expression levels of signature miRNAs in **(A)** diagnosing and **(B)** predicting transplant rejection.

miRNA analysis revealed a significant increase in the plasma expression of all three studied miRNAs (miRNA-155-5p, miRNA-122-5p, and miRNA-181a-5p) across all visits in the rejection group with respect to the nonrejection group. (**Table 3**) The AUROC analysis (**Table 3**) demonstrated that all three miRNAs exhibited high discriminatory power for both the diagnosis and prediction of liver rejection. In terms of diagnostic performance, miRNA-155-5p and miRNA-181a-5p showed identical AUC values (0.962, 95% CI: 0.943–0.981), while miRNA-122-5p also exhibited a strong diagnostic performance with an AUC of 0.907 (95% CI: 0.870–0.943). In terms of predictive capability, miRNA-155-5p achieved the highest AUC (0.870, 95% CI: 0.799–0.941), followed by miRNA-181a-5p (0.806, 95% CI: 0.716–0.897) and miRNA-122-5p (0.797, 95% CI: 0.725–0.869). The expression of miRNA-155p presented the best AUROC, as previously established in other cohorts (**Figure 6**).

**Table 3:** AUC curves for the expression levels of each of the miRNAs for both rejection diagnosis and prediction.

	Biomarker	AUC	Lower limit	Upper limit
DIAGNOSIS	miRNA-155-5p	0.962	0.943	0.981
	miRNA-122-5p	0.907	0.870	0.943
	miRNA-181a-5p	0.962	0.943	0.981
PREDICTIVE	miRNA-155-5p	0.87	0.799	0.941
	miRNA-122-5p	0.797	0.725	0.869
	miRNA-181a-5p	0.806	0.716	0.897

### 3.5 Combination of %dd-cfDNA and miRNA

We performed logistic regression to examine the contribution of each biomarker evaluated in this study. Logistic regression analysis (**Table 4**) revealed that %dd-cfDNA, miRNA-155-5p, and miRNA-181a-5p were significantly associated with liver rejection. %dd-cfDNA showed a positive coefficient (B = 0.062, p = 0.002), with an odds ratio of 1.064 (95% CI: 1.023–1.106). Similarly, miRNA-155-5p (B = 0.506, p = 0.005) and miRNA-181a-5p (B = 0.326, p = 0.012) exhibited significant associations, with odds ratios of 1.658 (95% CI: 1.161–2.368) and 1.386 (95% CI: 1.076–1.785), respectively. In contrast, miRNA-122-5p did not show a statistically significant association (p = 0.413). The model’s intercept was negative (B = -5.314, p < 0.001), indicating the baseline probability of rejection in the absence of the included biomarkers.

**Table 4:** Results of logistic regression analysis, including the estimated coefficients (B), odds ratios (Exp(B)), confidence intervals (CIs), degrees of freedom (Df) and p values (Sig) for the variables included in the model.

	B	Standard Error	Df	Sig.	Exp (B)	CI95 lower	CI95 upper
%dd-cfDNA	0.062	0.020	1	**0.002**	1.064	1.023	1.106
miRNA-155-5p	0.506	0.182	1	**0.005**	1.658	1.161	2.368
miRNA-122-5p	0.013	0.016	1	0.413	1.013	0.982	1.047
MiRNA-181a-5p	0.326	0.129	1	**0.012**	1.386	1.076	1.785
Constant	-5.314	0.649	1	<0.001	0.005		

LOGIT = −5.314 + 0.506·[miR-155-p level]+0.326·[miR-181a-5p level]+0.062·%dd-cfDNA.

Bold values are significant one (p<0.05).

## 4 Discussion

Our results demonstrated that longitudinal monitoring of dd-cfDNA levels is a highly valuable diagnostic method not only for detecting transplant rejection but also for identifying other clinical events that may impact liver graft functionality and evolution. Notably, we established a logistic regression model combining dd-cfDNA levels and miRNA expression levels, providing complementary information that may be useful in the diagnostic assessment of liver rejection.

Although one of the objectives of the study was to develop a diagnostic score for the assessment of rejection risk by combining miRNAs and dd-cfDNA, our analyses did not show an improvement in performance compared to the previously developed score by our group (based on miRNAs and chemokines). This may be explained by the fact that dd-cfDNA, as discussed in the manuscript, usually requires 2 to 4 weeks to return to baseline levels after liver transplantation. In our cohort, and in agreement with other studies, 85% of rejection episodes occurred within the first 4 weeks post-transplant, a period during which dd-cfDNA may still be elevated (>9.88%) due to peri-transplant factors. Together with the relatively small sample size of our study and the already strong performance of the previous score (AUROC=0.99 for diagnosis (87.5% sensitivity, 99.5% specificity, 91.3% PPV; 99.3% NPV; 98.9%)), these factors may account for the lack of additional benefit observed when dd-cfDNA was included.

Furthermore, dd-cfDNA levels were significantly elevated in patients with biliary stenosis; to our knowledge, this is the first study to report this finding in the context of liver transplantation. This diagnostic performance of dd-cfDNA levels can facilitate tailored adjustments to IST on the basis of the individual patient’s profile and enable the administration of targeted treatments. For example, antiviral therapy can be promptly initiated for patients with active CMV infections, offering a more personalized approach to posttransplant care.

In our cohort of stable functioning patients, the variability of dd-cfDNA values progressively decreased over successive visits, suggesting that any elevations in dd-cfDNA levels during follow-up beyond the first week would be readily detectable. This finding is supported by the observation that stable patients consistently maintained dd-cfDNA concentrations within a relatively narrow range, as reflected by the interquartile range (IQR).

In this study, we established a %dd-cfDNA cut-off value of 9.88% for diagnosing rejection, which is very similar to the results reported by CareDx (cut-off of 10%), which were obtained using the same technology. Moreover, these findings align with those from other studies (e.g., Kanamori et al., who reported a cut-off of 8.1%; Jana et al., who reported a cut-off of 10.2%; and Levitsky et al., who reported a cut-off of 5.3%). Considering the variability in methodologies across these studies, these results can be considered concordant. Notably, this cut-off value is significantly higher compared to those reported for other types of transplants, such as kidney (approximately 0.5%) or heart (approximately 0.25%) transplants (14, 16, 46–49). This difference is primarily due to the larger size and greater cellular turnover of the liver and the significant initial injury associated with transplantation of the organ (50).

As mentioned in the Introduction, there are two ways to express dd-cfDNA results: in absolute terms (copies/ml) and in relative terms (%). In this study, we used only the relative value, as this is the only method by which the technology employed in our laboratory reports the dd-cfDNA expression level. Currently, there is no clear consensus on which method of representation is optimal, as each has its own advantages and disadvantages, and different studies have reported conflicting results regarding the best way to express this biomarker. Jana K et al. (51) identified superior outcomes with the percentage fraction of dd-cfDNA than with the absolute dd-cfDNA value. However, other studies have argued that the most effective approach is to utilize both parameters to represent this biomarker (25). However, our goal is for this biomarker to be used in clinical practice within a short period, making economic viability a crucial factor. Providing the same result in two different forms via two different technologies significantly increases the associated costs.

dd-cfDNA levels are a reliable biomarker for diagnosing rejection, particularly because of their high sensitivity and NPV, which allows the avoidance of unnecessary biopsies by reliably ruling out patients who are not experiencing liver rejection. The only visit where we did not observe significant differences was the first visit, which corresponds to the first day posttransplantation. At this time, the overall transplantation process creates a substantially inflammatory environment, leading to alterations in most biomarkers. However, from the second visit (day 7) onwards, we observed significant differences in %dd-cfDNA between the rejection group and the stable group.

In our study, all transplants were DDLTs, which could explain the elevated dd-cfDNA levels observed at the initial visits. dd-cfDNA is released into the bloodstream in response to graft injury (32, 52). However, immediate posttransplantation elevations in this biomarker have been attributed primarily to IRI (53, 54). Studies have suggested that initial dd-cfDNA levels may be higher in DDLT than in living-donor liver transplantation (LDLT) because of prolonged cold ischemia times and graft-related adverse factors, such as macrosteatosis (51).

Nevertheless, one of the key advantages of the dd-cfDNA level lies in its practical applicability across diverse clinical settings, enabling longitudinal serial monitoring of graft health at any time without requiring a donor sample in both DDLT and LDLT.

Regarding potential confounding factors among the various events associated with graft dysfunction studied in our cohort, biliary stenosis appear to be the only condition that could potentially act as a confounder in the context of hepatic rejection. In contrast, no significant differences in dd-cfDNA levels were observed between stable patients and those with CMV infection, IRI, or nonspecific inflammation, suggesting that these events are unlikely to confound the interpretation of dd-cfDNA elevations related to rejection.

The fact that biliary stenosis was associated with increased dd-cfDNA levels could be explained by the possibility that some patients with biliary complications develop episodes of cholangitis, characterized by infection or inflammation of the biliary tract, which may result in hepatic inflammation and, consequently, elevated dd-cfDNA release. This hypothesis is supported by the observation that many of these patients exhibited a biochemical profile consistent with mixed cholestasis and cytolysis, including mild to moderate elevations in transaminases. However, this finding should be confirmed in a larger cohort with a sufficient number of patients in each clinical event subgroup. Nevertheless, combining %dd-cfDNA with miRNAs allows us to differentiate patients with rejection and those with biliary stenosis. For example, in patient 33 (**Figure 4**), despite the increase in dd-cfDNA levels during biliary stenosis, the expression levels of miRNA-155 and miRNA-181 remained low, which would not be the case in rejection, where the expression levels of these biomarkers are elevated. However, the expression of miRNA-122 was also elevated during cholestasis, since it is a liver-specific miRNA and liver damage also occurs in cholestasis.

In our cohort, the percentage of dd-cfDNA did not serve as a predictive biomarker for acute rejection; however, it can be used diagnostically. Therefore, for the prediction of acute rejection during the first weeks after LT, it is more advisable to utilize the miRNA signature proposed in this study. Regarding the timing of diagnosis, although the combination of miRNA and dd-cfDNA does not provide increased sensitivity or specificity in diagnosing acute rejection, it would be beneficial for confirming and discerning the underlying cause of liver dysfunction. As we have observed, the percentage of dd-cfDNA is significantly elevated only in cases of TCMR and biliary stenosis, whereas it is not significantly elevated in patients with other clinical events, such as CMV infection.

PCRR is a subtype of rejection characterized by the predominance of plasma cells in the graft biopsy. The low levels of dd-cfDNA in these cases can be explained by the pathogenesis of this type of rejection. PCRR is characterized by an immune response that is mediated mainly by plasma cells and lymphocytes that infiltrate the graft; however, this response tends to be more localized and less destructive at the cellular level than in other types of rejection. Consequently, there is less tissue damage and, therefore, reduced release of dd-cfDNA. Additionally, plasma cells may induce damage through the production of cytokines or inflammatory mediators, which does not necessarily lead to the apoptosis or necrosis of graft cells. As a result, the release of dd-cfDNA is further reduced with respect to other types of rejection, which are characterized by relatively extensive cellular destruction (55).

Leukopenia and leukocytosis have been reported to affect the dd-cfDNA value levels, resulting in falsely elevated or decreased values, respectively (19). In this study, all patients with leukopenia or leukocytosis were reviewed, and a comparison of their other laboratory results and clinical events suggested that these conditions did not significantly influence the results.

Despite not having any reported clinical events, some patients had dd-cfDNA levels above the cut-off of 9.88%. One possible explanation for this finding is that these patients may have had inflammation or minor liver damage that was undiagnosed due to the lack of biopsy. For example, some of these patients may have been consuming nonprescribed substances or medications with potential hepatotoxic effects.

## 5 Limitations of this study

Some limitations of this study should be acknowledged. First, repeated dd-cfDNA measurements were evaluated as a longitudinal biomarker for the occurrence, recovery, or progression of rejection episodes only in a limited group of patients, namely, those with clinical events such as rejections or other episodes of GD. This limited sample size may have affected the generalizability of our findings, and a larger cohort could provide more robust insights into the utility of dd-cfDNA for diagnosing these events.

Furthermore, the period over which this study was conducted allowed assessments of the biomarkers, primarily during early rejection episodes. Importantly, however, dd-cfDNA could be particularly valuable during the maintenance phase after the first year following liver transplantation. In this phase, dd-cfDNA could be used to identify subclinical rejections or chronic rejection processes. An increase in the levels of this biomarker is expected in patients experiencing inflammatory processes, particularly when alloreactivity is reactivated. Therefore, future studies should aim to include a cohort of patients over a longer posttransplant period to further investigate the utility of dd-cfDNA in detecting late-onset rejections and chronic GD.

dd-cfDNA has good characteristics as a biomarker for monitoring liver rejection; however, this type of acute rejection typically occurs during the first weeks posttransplantation, during which the %dd-cfDNA value is also elevated in patients without a risk of rejection. Therefore, we believe the utility of this biomarker would be best suited for monthly patient monitoring with a simple blood test starting two weeks posttransplant, during which the biomarker shows better efficacy. For the early posttransplant weeks, we recommend the use of miRNAs, which can both predict and diagnose acute rejection. The absence of elevated levels of dd-cfDNA and signature miRNAs, along with normal liver function tests, could prevent many biopsies in the future.

## Data Availability

The original contributions presented in the study are included in the article/**Supplementary Material**. Further inquiries can be directed to the corresponding author.

## Supplementary material

Supplementary Figure 1 Monitoring of donor-derived cell-free DNA (dd-cfDNA) levels in three transplant recipients (Patient 22, Patient 26, and Patient 49) over the first year post-transplant. Green arrows indicate time points of clinically confirmed acute rejection episodes. Each line represents the dynamic changes in %dd-cfDNA at predefined time points.
