# Supplementary figures and images for: Donor-derived cell-free DNA and miRNA monitoring for the early prediction and diagnosis of liver allograft rejection and patient outcomes
Source: Front Immunol. 2025 Jun 24;16:1604200. doi: 10.3389/fimmu.2025.1604200 (PMC12234302; doi:10.3389/fimmu.2025.1604200)

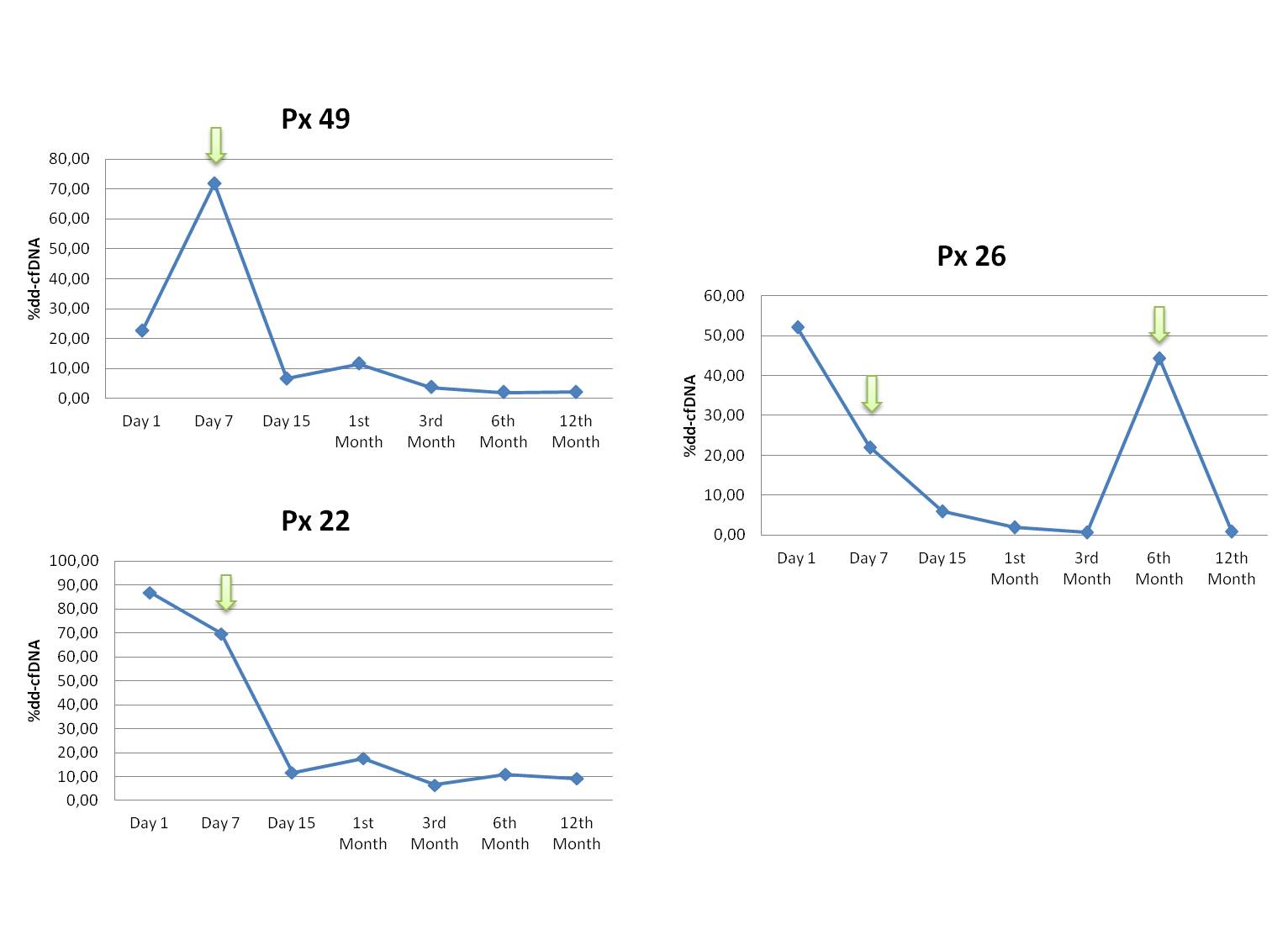

Supplement: Supplementary Figure 1 — Monitoring of donor-derived cell-free DNA (dd-cfDNA) levels in three transplant recipients (Patient 22, Patient 26, and Patient 49) over the first year post-transplant. Green arrows indicate time points of clinically confirmed acute rejection episodes. Each line represents the dynamic changes in %dd-cfDNA at predefined time points. [file Image1.jpeg]
